# T137. CLASSIFICATION OF RECENT-ONSET PSYCHOSIS BASED ON RESTING-STATE FUNCTIONAL CONNECTIVITY AND THE RELATIONSHIP TO NEUROCOGNITIVE IMPAIRMENT

**DOI:** 10.1093/schbul/sby016.413

**Published:** 2018-04-01

**Authors:** Johanna Weiske, Anne Ruef, Shalaila Haas, Carolina Bonivento, Nikolaos Koutsouleris, Lana Kambeitz-Ilankovic

**Affiliations:** 1 Ludwig Maximilians University, University Psychiatric Hospital; 2 University of Udine

## Abstract

**Background:**

Impairments in cognitive functioning are a core feature of psychotic disorders and they have been associated with resting-state functional connectivity (rsFC) alterations in patients suffering from psychosis (Dauverman et al., 2014). Multivariate pattern analysis (MVPA) has proven to be a useful tool in the investigation of rsFC alteration in psychosis and in detecting subtle differences in multidimensional data sets (Kambeitz et al., 2015). In this study, we differentiated recent-onset psychosis patients (ROP) from healthy controls (HC) using a Support Vector Machine (SVM) classification based on rsFC. Furthermore, we investigated the relationship of the discriminative rsFC pattern to neurocognitive measures.

**Methods:**

Resting-state fMRI and neurocognitive measures were obtained from 220 HC and 115 ROP across 7 sites of the PRONIA consortium. The rsFC matrix was estimated for each subject by calculating pairwise correlations between mean time series of 90 brain regions based on AAL parcellation. A L1-regularized L2-loss SVM was trained to classify ROP vs. HC based on rsFC in a repeated nested cross-validation. Decision scores for ROP were correlated with cognitive measures derived from the following neuropsychological tests: Rey Auditory Verbal Learning Task (RAVLT), Phonetic and Semantic Verbal Fluency, Diagnostic Analysis of Nonverbal Accuracy, Forward and Backward Digit Span, Self-ordered Pointing Task, and Salience Attribution Test.

**Results:**

The classification algorithm was able to differentiate ROP and HC with a balanced accuracy (BAC) of 71.3% based on rsFC. The discriminative connectivity pattern included short-range connections between left putamen and left hippocampus, right putamen and right caudate nucleus, left superior frontal and right inferior orbitofrontal regions, as well as long-range connections between left and right occipital cortex and left cingulate gyrus, left supramarginal gyrus and right temporal pole. Two negative correlations between the SVM decision scores for ROP and measures of the RAVLT were significant (delayed recall: r=-0.3, Bonferroni –adjusted p<.04; recall after interference: r=-0.3, Bonferroni-adjusted p<.02).

**Discussion:**

The classification performance was driven by a rsFC pattern including areas involved in memory processing, such as hippocampus and cingulate gyrus (Allen et al., 2007) as well as regions related to language processing, such as the supra marginal gyrus (Li et al., 2009). The negative correlation of rsFC-based decision scores with RAVLT measures shows that patients whose verbal learning and memory is more severely impaired exhibit a more distinctive rsFC pattern than patients with less impaired verbal memory.

